# Behavioral and brain pattern differences between acting and observing in an auditory task

**DOI:** 10.1186/1744-9081-5-5

**Published:** 2009-01-20

**Authors:** Irene S Karanasiou, Charalabos Papageorgiou, Eleni I Tsianaka, George K Matsopoulos, Errikos M Ventouras, Nikolaos K Uzunoglu

**Affiliations:** 1 Institute of Communications and Computer Systems, National Technical University of Athens, 9, Iroon Polytechneiou str., 157 73 Zografou Campus, Athens, Greece; 2 School of Medicine, National and Kapodistrian University of Athens, Greece; 3 Technological Education Institution of Athens, Greece

## Abstract

**Background:**

Recent research has shown that errors seem to influence the patterns of brain activity. Additionally current notions support the idea that similar brain mechanisms are activated during acting and observing. The aim of the present study was to examine the patterns of brain activity of actors and observers elicited upon receiving feedback information of the actor's response.

**Methods:**

The task used in the present research was an auditory identification task that included both acting and observing settings, ensuring concurrent ERP measurements of both participants. The performance of the participants was investigated in conditions of varying complexity. ERP data were analyzed with regards to the conditions of acting and observing in conjunction to correct and erroneous responses.

**Results:**

The obtained results showed that the complexity induced by cue dissimilarity between trials was a demodulating factor leading to poorer performance. The electrophysiological results suggest that feedback information results in different intensities of the ERP patterns of observers and actors depending on whether the actor had made an error or not. The LORETA source localization method yielded significantly larger electrical activity in the supplementary motor area (Brodmann area 6), the posterior cingulate gyrus (Brodmann area 31/23) and the parietal lobe (Precuneus/Brodmann area 7/5).

**Conclusion:**

These findings suggest that feedback information has a different effect on the intensities of the ERP patterns of actors and observers depending on whether the actor committed an error. Certain neural systems, including medial frontal area, posterior cingulate gyrus and precuneus may mediate these modulating effects. Further research is needed to elucidate in more detail the neuroanatomical and neuropsychological substrates of these systems.

## Background

The ability to monitor ongoing performance is critical to behavioral adaptation in changing environmental settings. Especially, the monitoring of performance errors serves to optimize future response behavior.

The neural basis of error processing has attracted great interest, because its elucidation promises a better understanding of the mechanisms underlying adaptive and non-adaptive behavior. The strongest evidence for the existence of the neural system that implements error processing has come from the detection of the error-related negativity (ERN), a component of the event-related potentials (ERPs) generated within the medial-frontal cortex that is sensitive to error in performance [1,2].

This class of ERP components comprises a growing list of potentials, including the ERN/Ne elicited by error responses in reaction time tasks [1,3], the N2-like potentials elicited by error feedback stimuli [4-6], the variety of N2-like potentials elicited in situations of response conflict or response inhibition [7-9]. Importantly, according to the conflict hypothesis [10,11], which states that the Ne does not reflect the mismatch, but rather the conflict between response representations, knowledge about the correct response is not necessary and the error related potential is not specific for errors, but depends only on the amount of conflict.

In this context it should be noted that based on the Mirror Neuron System MNS hypothesis [12], which suggests that acting and observing are associated with similar neural mechanisms, the feedback ERN has been studied in two participant conditions [13-16]. These studies focused on the way this type of ERN reflects the evaluation of outcomes induced by others [14,16] even those of a simulated Brain Computer Interface BCI [15]. The feedback-related negativity-like effects were obtained in both single and two-participant condition, suggesting that similar neural mechanisms are involved in evaluating the outcomes of one's own and the other's actions.

Up to date the exact mechanisms underlying the error system continue to be a subject of ongoing debate, since their patterns and specificity still remain poorly understood. In this sense it is interesting to study potential differences in the pattern of activity elicited by observed errors than from self-made errors in conditions where the roles of acting and observing are alternating.

Considering the aforementioned viewpoint as well as findings of recent experimental studies that there is an ongoing debate about the characterization of brain activity correlated with errors, a simple task design was used to compare the electrophysiological effects related to observed and self-made errors during performance monitoring in two-participant conditions of varying complexity. The electrophysiological recordings were simultaneously performed for both subjects as they alternatively exchanged roles between acting and observing. With synchronous two-subject recordings, comparisons of the elicited ERP conventional parameters (amplitudes, latencies), could be made both between actors and observers as well as between errors and non-errors. Our research hypothesis was to study the ERP patterns of observers and actors in a two-participant experiment elicited upon receiving feedback information of the actor's response.

The task used in the present research was an auditory identification task comprising one single-actor and two two-actor conditions. In the latter the participants performed the task in an alternating fashion, exchanging roles between acting and observing in each trial. The aim for the actor is to correctly map a horizontal slider position onto an active tone-frequency range and in each trial he/she selects a slider position that matches a tone that is initially presented to him/her. Both participants receive feedback information corresponding to the slider position selection. The frequency range in which the auditory cues/stimuli are presented varies in the experimental conditions in order to explore the participants' competency. In one of the two-actor conditions, auditory stimuli from the same frequency range are presented to both participants while in the other condition each participant is presented with tones from his\her individual frequency range that is different from that of the partner. The hypothesis is that cue frequency dissimilarity may hinder the pitch identification process by increasing the complexity of the task. In the case where the actors received different tones from the observers, this would impact performance and how accuracy is judged. The subsequent neural responses to feedback are investigated in all conditions and in both actors and observers.

The recordings of the scalp ERPs both for the actor and the observer were simultaneous. We investigated the error related component of the ERPs related to feedback information from the actor response recorded during acting and observing both with the conventional constituents (amplitudes and latencies) as well as with the LORETA source localization method which differentiates between structural and energetic processes related to information processing as revealed by the associated ERP waveform [17-19].

## Methods

### Participants

Fourteen healthy individuals (eight men and six women), with mean age of 26.6 ± 2.9 years and high level education (education years 17.7± 2.3), all with normal hearing as measured by pure-tone audiograms (thresholds <15 dB HL), participated in the experiment. The male and female subgroups were homogeneous with regards to age and educational level. All the participants were right-handed and had no history of any hearing problem. Informed consent was obtained from all subjects.

### Stimuli and procedures

In the present research an auditory identification task has been used in one single-actor and two two-actor conditions. In the two-actor or "Joint" conditions the participants performed the task in an alternating fashion, meaning that when one subject performed the task (actor) the other observed (observer) whereas in the Single condition performed the task alone. From one trial to the next, the roles of actor and observer were switched between the two dyad members.

The frequency range in which the auditory cues were presented varied in the experimental conditions. In one of the joint conditions, auditory stimuli from the same frequency range were presented to both participants (Joint-1) while in the other condition each participant was presented with tones from different frequency ranges (Joint-2). All participants were examined in all three conditions (single, Joint-1, Joint-2) in varying order. The dyads in the Joint-1 and Joint-2 condition were the same.

The actor and observer sat opposite but were screened from each other. They both had computer screens in front of them. At each trial they both heard the stimulus tone with duration of 1 sec presented through the headphones. The stimulus tone was randomly selected for each trial within the fixed frequency range. The actor's task was to position a slider presented on the computer screen with a gamepad such that the slider position would match the frequency of the stimulus tone. The mapping of the frequency range was fixed within a block of trials; however, at the start of the trial blocks, the participants did not know the scaling of the frequency range within which the slider position should be mapped. After the positioning of the slider by the actor, the frequency corresponding to the actor's selected slider position (Feedback tone) was presented to both the actor and the observer.

The single condition consisted of 40 trials. The joint conditions consisted of 80 trials for both subjects. The trials in the joint conditions were accomplished alternately by the two participants, 40 trials for each participant. The stimuli were presented from four frequency ranges with a bandwidth 400-Hz namely Range 1: 200–600 Hz, Range 2: 620–1020 Hz, Range 3: 1040–1440 Hz, Range 4: 1460–1860 Hz.

Before the experiment, the subjects were submitted to an acoustic pre-test in order to examine their hearing ability in the four frequency ranges that were used in the experiment. During this test two tones from each range were presented to the participants. Then, the participants had to identify which of these tones was higher than the other. The frequencies of the two tones selected from each range for the acoustic test were determined as the 25% and the 75% of each range of 400 Hz bandwidth. The subjects heard the tones with their headphones and responded orally to the experimenter. All participants were capable to discriminate between the tones presented in the pre-test.

### EEG recordings and experimental setup

The experimental setup included a Faraday room, which screened any electromagnetic interference that could affect the measurements. The EEG was recorded continuously using a 32-channel electrode cap (Biosemi, Active-two System) according to the International 10–20 EEG system [20]. The electrodes used were Fp1, Fp2, Pz, Fz, O1, O2, P3, P4, P7, P8, C3, C4, T7, T8, F3, F4, F7, F8, Cz, Oz, CP5, CP6, CP1, CP2, FC1, FC2, FC5, FC6, AF3, AF4, PO3 and PO4.

The bioelectrical brain activity was simultaneously recorded from both participants using two different recording systems, daisy chained in a master-slave relationship. Galvanic isolation of both participants was ensured by using optical receiver for trigger inputs, while in parallel, interference pickup was also eliminated. The electrode cables were also bundled to eliminate potential magnetic interference. The vertical electro-oculogram (EOG) was recorded bipolarly from electrodes placed above and below the eyes and the horizontal EOG was monitored from electrodes at the outer canthi of the eyes. The data were filtered off-line, high-pass at 0.05 Hz and low-pass at 35 Hz. All signals were digitized with a sampling rate of 256 Hz. All scalp signals were referenced online to both mastoids, but were later offline re-referenced to the average of all scalp electrodes. Trials were averaged to ERPs separately for each condition and each subject, relative to a 100 ms pre-stimulus (Feedback tone) baseline.

To eliminate EOG artifact, trials with EEG voltages exceeding 80 μV were rejected from the average. Artifact rejection and averaging were performed offline. Due to artifact contaminated epochs the measurements of one participant dyad were excluded. Consequently, the data were analyzed for 6 dyads.

### Categorization of correct and erroneous responses

The ability of each participant to differentiate tones and his/her auditory frequency perception resolution can be described in terms of an Equivalent Rectangular Bandwidth (ERB) as a function of a centre frequency, which better represents auditory frequency selectivity according to psychoacoustics. In the present context the concept of ERB can be conceived as the acceptable bandwidth around the stimulus frequency within which the response frequency can be considered as a correct response value. According to psychoacoustics theory [21-24], the Equivalent Rectangular Bandwidth (in Hz) can be approximated according to the following formula: *B*_*e *_= 6.23 10^-6 ^*f *^2 ^+ 9.339 10^-2 ^*f *+ 28.52. The discrimination between the actor's erroneous and non-erroneous responses was performed with the use of the above formula.

### LORETA source localization method

The low resolution brain electromagnetic tomography (LORETA) differentiates between structural and energetic processes related to information processing as revealed by the associated EEG/ERP waveform [17,18]. The structural level, revealed by the location of the local maxima of the current source density distribution, describes the time dependent network of activated brain areas. The magnitude of the source strength, a measure of the energetic component, describes the allocation of processing resources [19]. The utilized LORETA version was registered to the Talairach brain atlas [25]. The solution space consisted of 2394 voxels with a spatial resolution of 7 mm. Average LORETA images were constructed across all subjects in all 4 cases (both for actors and observers depending on whether the actor committed an error or not). The voxel-by-voxel pairwise t-test differences were carried out for the observers depending whether the actor made an error or not. The structure-Probability Maps atlas [26] was used to identify which brain regions were involved in the ERP waveforms as well as in differences between the compared groups. Brodmann area(s) and brain regions close to the observed locations identified by the Talairach coordinates are reported [25].

### Statistical analysis

The primary behavioral outcome variable was the Absolute Frequency Error (AFE), defined as the absolute difference between the stimulus and response frequency. A univariate general linear model (GLM) was used with the absolute frequency error as the dependent variable and Trial (n = 40), Condition (Single, Joint-1, Joint-2), Frequency Range (n = 4) and Order (n = 3) as fixed factors.

The time window within which ERP analysis was performed was from -200 msec to +500 msec around the Feedback tone, which included 180 time points. In order to take into account neurophysiological processes that took place between Stimulus and Feedback tone that could affect the elicited ERP waveforms, the analysis was also performed by selecting a100 ms baseline before the Stimulus tone yielding the same results. The mean amplitude values for every subject at each time point were subjected to three-way ANOVA. The first factor was whether the subject was the actor or the observer. The second factor was whether the actor had committed an error or not, while the third factor was the condition (Joint-1, Joint-2). Factor or interaction effects were considered significant only if they occurred over a time window of at least 20 msec. When significant factor or interaction effects were found, post hoc analyses with Bonferroni corrections were carried out. Statistical significance was set at 0.05.

The LORETA mappings were performed at the time window 144–171 msec where significant differences were observed resulting from the ANOVA procedures.

## Results

### Behavioral data

**Figure 1:**
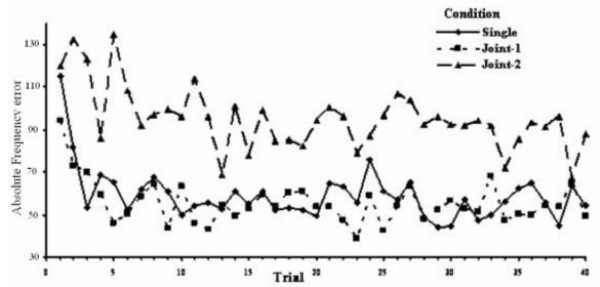
**Absolute frequency error**. Average absolute frequency error (Hz) for the three conditions as a function of trial number.

The ANOVA procedure with AFE as the dependent variable and Condition, Trial, Frequency Range and Order as the independent factors revealed that only Condition (F = 23.6, p < 0.01) and Trial (F = 3.1, p < 0.01) had a significant effect. However the significance of the Trial effect is only partial. Indeed, as post-hoc contrasts confirm, only the first trial (and partly the second one) had a significantly larger AFE than the other trials. Accordingly the significance of the Trial effect, after having excluded the data of the first two trials, disappeared. Consequently, the factor responsible for most of the variability of AFE remained the Condition (F = 28.5, p < 0.01). The average AFE absolute frequency error (Hz) for the three conditions as a function of trial number is depicted in Figure 1, which clearly depicts that the subjects' performance is significantly worse in the Joint-2 condition throughout the forty trials, with the exception of the first one, while the Joint-1 condition was practically the same as the Single condition. It is interesting to note that the overall mean AFE values in the Joint-2 condition do not differ from the mean AFE values of the first trial of the Single and Joint-1 conditions.

### Event related potentials data

The ANOVA procedures with the amplitudes at each lead and each time point with Condition, Response Category (Erroneous, Correct) and Subject Status (Actor, Observer) revealed that condition did not have any significant effect, either by itself or through its interactions with the other two factors. Therefore subsequent analyses of the amplitudes were performed disregarding the condition. Consequently, it should be noted that in the final ERP analysis the data from both Joint conditions were used, resulting in the employment of 80 trials per acting and observing states. The final ANOVA revealed that for two electrodes, CP1 and F4, there was a time window 144–171 msec, where a continuous Response Category × Subject Status interaction was found to be significant.

**Figure 2:**
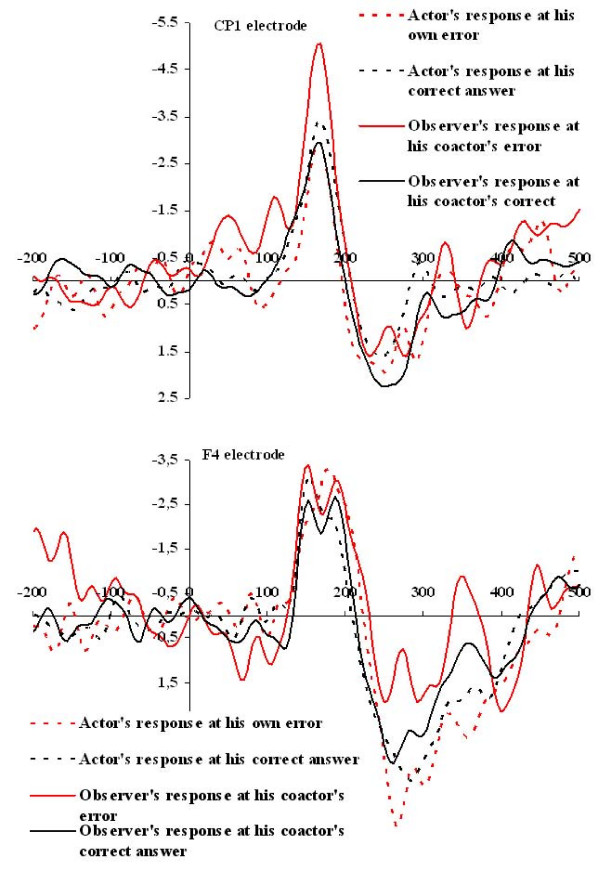
**Mean amplitude values at CP1 and F4 electrodes around the Feedback tone**. Mean amplitude values at the CP1 (top) and F4 (bottom) electrode around the Feedback tone for the actors and the observers depending on whether the actor had committed an error.

The nature of these interaction effects can be understood by examining Figure 2, where the mean amplitude values at the CP1 and F4 electrodes around the Feedback tone for the actors and the observers depending on whether the actor had committed an error are shown. The top part of the Figure 2 clearly depicts an unambiguous negative peak at the CP1 electrode around the time point of 167 msec both for the actors and the observers, at both correct and erroneous answers of the actor. However the height of this peak differs significantly depending on whether the subject was the actor or the observer in conjunction with whether the actor had committed an error. Post-hoc pairwise comparisons revealed that at the time window 144–171 msec the observers' amplitudes at the CP1 electrode were significantly greater when their co-actor had committed an error (continuous red line) than when their co-actor had not made an error (continuous black line). At the same time window when the actors had committed an error the observers had significantly greater amplitudes (continuous red line) than the actors (dotted red line). Conversely no differences were observed between the mean amplitudes of the actors and the observers when the actors had answered correctly, or of the actors for their correct and non-correct responses.

The bottom part of the Figure 2 depicts the same amplitude values at the F4 electrode. The first negative peak is observed at an earlier latency value of 152 msec. Nonetheless, as the figure clearly shows, the amplitudes follow the same patterns of differences, regarding the Response × Status interaction effect, with those described for the CP1 electrode, although in this case the contrasts are not so sharp.

It is interesting to note that the LORETA solution of the activation patterns in the time window of 144–171 msec after the onset of the Feedback tone for the actors and the observers depending on whether the actor had committed an error produced similar results for all four cases. In all cases the voxel of maximum activation is at Brodmann area 31, Cingulate Gyrus, Limbic Lobe. The most notable brain areas with high intensity of the density function are shown in Table 1. This finding corroborates the above sited results for the scalp electrodes, showing that activation for the four different states is practically the same and it differs only in intensity. This validation assumes a more general character, since the LORETA inverse solution takes into consideration the amplitude values of all the scalp electrodes.

**Table 1:** Brain areas of maximal activation

Brodmann area 31, Cingulate Gyrus, Limbic Lobe
Brodmann area 7, Precuneus, Parietal Lobe

Brodmann area 5, Paracentral Lobule, Frontal Lobe

Brodmann area 23, Cingulate Gyrus, Limbic Lobe

Brodmann area 6, Paracentral Lobule, Frontal Lobe

Brain areas of maximal activation in the time window of 144–171 msec after the onset of the Feedback tone

**Figure 3:**
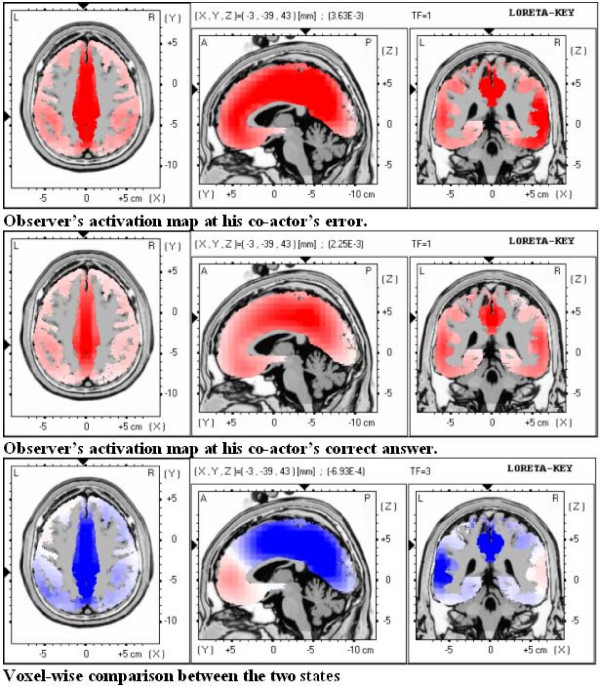
**LORETA maps of the activation patterns and differences**. LORETA solution of the activation patterns in the time window of 144–171 msec after the onset of the Feedback tone for the observers at their co-actor's errors and correct answers and voxel-wise comparison between the two states.

Moreover the voxel-wise LORETA comparisons proved that maximal differences between different states are located at the voxels of maximum activation. This is exemplified in Figure 3. The first two LORETA diagrams show the observers' activation maps at their co-actors' errors and correct answers. Clearly, the activation maps are similar but different in intensity. Specifically at the voxel of maximum activation (Brodmann area 31 Cingulate Gyrus, Limbic Lobe), the intensity of the density function for the observer's response at his co-actor's error was 3.65 × 10^-3^, while for the observer's response at his co-actors correct answer the intensity was 2.25 × 10^-3^. The intensities for the actor (not shown in the figure) at his own error and at his correct answer were 2.65 × 10^-3 ^and 2.55 × 10^-3 ^respectively, i.e. they lay intermediately between the observer's values, as was found for the electrodes. The bottom figure shows the result of the voxel, pairwise LORETA comparisons between the two states for the observers: when the actors were correct and when the actors were wrong. More intense blue color denotes larger differences between the two states. A comparison of this figure with the activation maps leads to the conclusion that the difference map practically coincide with the activation maps. Specifically the voxel of maximal differences is again at Brodmann area 31 Cingulate Gyrus, Limbic Lobe, at which the significance of the differences is <0.01.

In conclusion the activation patterns of the actors and the observers at the actor's errors and correct answers are temporally and spatially congruent, varying only in the intensity of the density function and maximal differences in the intensity between states are observed at the areas of maximum activation.

## Discussion

The present study was performed to examine brain activity in a two-participant condition task following actor-response related feedback correlated with errors. The behavioral results showed that the participants had significantly poorer performance in conditions with increased complexity. Comparisons of the ERP measurements revealed that the subjects during the observing condition had a significantly greater negative deflection located at the medial frontal area and superior parietal regions when their co-actors committed an error than when they had correct responses. The LORETA source localization method yielded significantly larger electrical activity in the supplementary motor area (Brodmann area 6), the posterior cingulate gyrus (Brodmann area 31/23) and the parietal lobe (Precuneus/Brodmann area 7/5). Nevertheless, the condition factor did not have any significant effect on the ERP patterns of actors and observers.

Regarding the behavioural data, results showed that participants' performance was strongly influenced by cue dissimilarity yielding significantly poorer scores in the more complex Joint-2 condition. The Joint-1 condition yielded similar results with the Single condition. In the first trial, in all conditions, the actor is faced with the unknown. The participant has no measure of inference with regards to the minimum and maximum and hence to the range of his/her scale, so the first positioning of the slider is in essence arbitrary.

From the second trial onwards the actor works comparatively with the previous trial. The mapping of the tone frequency range is better achieved in the case when sound identification is assisted by sound discrimination, resulting from the comparison of the present with the previous tone. Such is the case of the Joint-1 and Single conditions, where the participants are provided stimuli from the same frequency range. As a result their task performance was significantly better than in the Joint-2 condition, where the participants were provided stimuli from different frequency ranges. Each participant, by observing the actions of his/her partner, who performs in a different frequency range than him/her, seems to demodulate his/her perception of the scale of his/her own frequency range, and results in being disoriented when trying to place the slider correctly as actor in the subsequent trial. By receiving distracting information from the previous cue not belonging to the mapped frequency range resulted in poor performance, which is practically the same as his/her first trial. In the Single and Joint-1 conditions the participants' answers are fine-tuned as the trials in a block evolved but each actor seemed to start right from the beginning during the Joint-2 condition throughout the trial blocks

The participants of each dyad were simultaneously recorded and the relevant ERP analysis was performed using both its conventional constituents (amplitude, latency) and the LORETA patterns. Regarding the analysis of amplitude data at the Feedback tone comparisons of measurements of conventional constituents of the relevant event related potential, revealed that at the time window 144–171 msec the observers' amplitudes at the F4 and CP1 electrodes were significantly greater when their co-actor had committed an error than when their co-actor had not made an erroneous response. These findings could be better understood if we take into account the postulates of the Attribution theory [27,28] which claims that when someone observes another person he/she is inclined to regard the other more responsible for the negative results than the positive ones or tends to assume that the other is more responsible for the lack of effort than inferiority or tends to consider the other more responsible for the negative outcomes than his/her own self.

The neural sources with the LORETA method yielded significant electrical activity in the supplementary motor area (Brodmann area 6), the posterior cingulate gyrus (Brodmann area 31/23) and the parietal lobe (Precuneus/Brodmann area 7/5).

A large number of electrophysiological and neuroimaging studies have shown that the fronto-parietal mirror neuron system is engaged during the observation of actions of others and of our environment [29-33]. The obtained results with regard the ERP patterns of the observer at the F4 lead and its source localization at the supplementary motor area (Brodmann area 6) are in accordance with previous studies, suggesting that ERPs related to error of the medial frontal area and its associated activation of the Brodmann area 6 is a part of an evaluative function signifying 'worse than expected events' [29,30,34].

Interestingly, Gehring and Willoughby [35], with the view to add to the knowledge whether all medial frontal negativities are created equal, observed that the feedback-related medial frontal negativities are somewhat more right lateralized and anterior in the scalp distribution than is the classic ERN. Topographic analysis can show the disparity between two ERP components: a difference in scalp topographies implies that the underlying configuration of neural generators must be different [36,37]. It is quite plausible they share some activity but that there is also some distinct activity contributing to one or both components. Although these results indicate that the ERN and the feedback-related negativities are not identical, it is still an open question whether the conflict-detection of the ERN must also accommodate findings from studies of the feedback negativities. Some or all of the generators of the ERN may be active when the feedback-related medial frontal negativity is recorded, and vice versa [35].

The finding, concerning the ERP located at the superior parietal lobe and the associated activation of regions consisting of the posterior cingulate gyrus (Brodmann area 31/23) and the parietal lobe (Precuneus/Brodmann area 7/5), seems to be in congruence with studies [29,38] proposing a distributed error processing system in the human brain including the medial prefrontal area, the anterior cingulated cortex, and the posterior cingulated/precuneus (Brodmann area 31//29). However, an alternative explanation concerning this finding- which does not exclude the above mentioned but possibly complements it- provides the hypothesis that holds that human error processing is hierarchically organized [39]. According to this approach, it seems likely that the error related negative deflection located at the superior parietal area in the present study may reflect the low-level error information [39].

Within this framework electrophysiological studies based on No/Go events [40] have suggested that activity of the parietal cortex reflects stored potential motor response to external inputs, while activity in the prefrontal cortex reflects the intended response. Furthermore, the precuneus is also transiently activated when external feedback shifts from correct to incorrect during tasks, where subjects are required to alter stimulus-response judgments [41]. Moreover, lesions in the parietal cortex are known to induce apraxia, an inability to manipulate common objects [42,43].

Finally, this finding seems to be consistent with studies showing activation of posterior cingulate and parietal areas during action observation [29,44]. These results support the notion that these brain areas form a network associated with spatial attention and motor intention [44-46].

In our hypothesis we expected that if the actors received different tones from the observers, this would impact the way accuracy is judged. The results show that indeed the participants showed significantly poorer performance in conditions with increased complexity. We also investigated the subsequent neural responses to feedback in all conditions for both actors and observers. The results revealed that the condition factor did not have any significant effect on the ERP amplitudes of actors and observers even when it was separately studied in single factor ANOVAs, not taking into consideration the error and non-error trials. This finding, indicates that the error and non-error elicited ERPs are independent of the condition and may be due to the fact that psychophysiological indices such as ERPs recorded in the current study, represent aspects of the 'endophenotype', while behavioral performance expresses the 'phenotype' part of the behavior. It is suggested that 'the endophenotype provides the manifestation of a disorder via anomalies not observable by diagnostic interviews or traditional psychological measures' [47].

## Limitations

Certain limitations of this investigation warrant consideration. Firstly, sample sizes were relatively small and the main findings need to be replicated in independent samples and it is to be determined, whether there is an association in a task-specific manner or across tasks.

Secondly, the age spectrum of the participants is referred to young adults; hence future studies controlling age, trait and state parameters in conjunction with more experiments that combine the time resolution of event-related potentials with the spatial resolution of brain imaging techniques may lead to clearer definitions of the brain functions in relation to the current findings.

## Conclusion

The obtained results showed that in an auditory identification task that included both acting and observing settings, cue dissimilarity between trials was a demodulating factor leading to poorer performance. Even though this increased complexity considerably impaired behavioral performance, it did not have any significant effect on the ERP patterns of actors and observers.

Additionally, the electrophysiological results suggest that feedback information results in different ERP patterns of observers and actors depending on whether the actor had made an error or not. Certain neural systems, including medial frontal area, posterior cingulate gyrus and precuneus may mediate these modulating effects. Further research is needed to elucidate in more detail the neuroanatomical and neuropsychological substrates of these systems. The finding of consistent activity in specific brain regions during the processing and evaluation of one's own and others' actions may have significant implications in clinical research.

## Competing interests

The authors declare that they have no competing interests.

## Authors' contributions

ISK and EIT performed the acquisition and analysis of the EEG data. CP, ISK and EIT participated to the interpretation of the results and composed the manuscript. GKM and EMV participated to the interpretation of the results. NKU conceived the core of the study design. NKU, GKM and EMV also revised the manuscript critically. All authors read and approved the final manuscript.

## References

[B1] Gehring WJ, Goss B, Coles MGH, Meyer DE, Donchin E (1993). A neural system for error detection and compensation. Psychol Sci.

[B2] Falkenstein M, Hohnsbein J, Hoormann J (1995). Event-related potential correlates of errors in reaction tasks. Electroencephalogr Clin Neurophysiol.

[B3] Falkenstein M, Hohnsbein J, Hoormann J, Clanke L (1991). Effects of cross-modal divided attention on late ERP components II. Error processing in choice reaction time. Electroencephalogr Clin Neurophysiol.

[B4] Miltner WHR, Braun CH, Coles MGH (1997). Event-related brain potentials following incorrect feedback in a time estimation task: evidence for a "Generic" neural system for error detection. J Cogn Neurosci.

[B5] Holroyd CB, Coles MG (2002). The neural basis of human error processing: reinforcement learning, dopamine, and the error-related negativity. Psychol Rev.

[B6] Ruchsow M, Grothe J, Spitzer M, Keifer M (2002). Human anterior cingulate cortex is activated by negative feedback: evidence from event-related potentials in a guessing task. Neurosci Lett.

[B7] Gehring WJ, Gratton G, Coles MGH, Donchin E (1992). Probability effects on stimulus evaluation and response processes. J Exp Psychol Hum Percept Perform.

[B8] Kopp B, Mattler U, Goertz R, Rist F (1996). N2, P3 and the lateralized readiness potential in a nogo task involving selective response priming. Electroencephalogr Clin Neurophysiol.

[B9] Falkenstein M, Hoormann J, Hohnsbein J (1999). ERP components in go/nogo tasks and their relation to inhibition. Acta Psychol.

[B10] Carter C, Braver T, Barch D, Botvinick M, Noll, Cohen J (1998). Anterior cingulate cortex, error detection, and the online monitoring of performance. Science.

[B11] Van Veen V, Carter C (2002). The timing of action-monitoring processes in the anterior cingulate cortex. J Cogn Neurosci.

[B12] Rizzolatti G, Fogassi L, Gallese V (2001). Neurophysiological mechanisms underlying the understanding and imitation of action. Nat Rev Neurosci.

[B13] Bates AlT, Patel TP, Liddle PF (2005). External behavior monitoring mirrors internal behavior monitoring. J Psychophysiol.

[B14] Yu R, Zhou X (2006). Brain responses to outcomes of one's own and other's performance in a gambling task. Neuroreport.

[B15] Ferrez PW, Millán JR (2008). Error-related EEG potentials generated during simulated brain-computer interaction. IEEE Trans Biomed Eng.

[B16] Itagaki S, Katayama J (2008). Self-relevant criteria determine the evaluation of outcomes induced by others. Neuroreport.

[B17] Pascual-Marqui RD, Michel CM, Lehmann D (1994). Low resolution electromagnetic tomography: a new method for localizing electrical activity in the brain. Int J Psychophysiol.

[B18] Michel CM, Thut G, Morand S, Khateb A, Pegna AJ, Grave de Peralta R, Gonzalez S, Seeck M, Landis T (2001). Electric source imaging of human brain functions. Brain Res Brain Res Rev.

[B19] Anderer P, Saletu B, Semlitsch HV, Pascual-Marqui RD (2002). Structural and energetic processes related to P300: LORETA findings in depression and effects of antidepressant drugs. Methods Find Exp Clin Pharmacol.

[B20] Jasper H (1958). The ten-twenty electrode system of the international federation. Electroencephalogr Clin Neurophysiol.

[B21] Patterson RD (1976). Auditory filter shapes derived with noise stimuli. J Acoust Soc Am.

[B22] Moore BCJ, Glasberg BR (1983). Suggested formulae for calculating auditory-filter bandwidths and excitation patterns. J Acoust Soc Am.

[B23] Glasberg BR, Moore BCJ (1990). Derivation of auditory filter shapes from notched-noise data. Hear Res.

[B24] Patterson R, Robinson K, Holdsworth J, Mc Keown D, Zhang C, Allerhand M, Cazals Y, Demany L, Horner K (1992). Complex sounds and auditory images. Proceedings of the 9 h International Symposium on Hearing, Auditory Physiology and Perception: 1991; Carcans, France.

[B25] Talairach P, Tournoux J (1988). A Stereotactic Coplanar Atlas of the Human Brain.

[B26] Lancaster JL, Woldorff MG, Parsons LM, Liotti M, Freitas CS, Rainey L, Kochunov PV, Nickerson D, Mikiten SA, Fox PT (2000). Automated Talairach Atlas labels for functional brain mapping. Hum Brain Mapp.

[B27] Rusbult CE, Van Lange PA (2003). Interdependence, interaction, and relationships. Annu Rev Psychol.

[B28] Johnson DW, Johnson RT (2005). New developments in social interdependence theory. Genet Soc Gen Psychol Monogr.

[B29] Herrmann MJ, Roemmler J, Ehlis A-C, Heidrich A, Fallgatter AJ (2004). Source localization of the error-related-negativity and positivity. Brain Res Cogn Brain Res.

[B30] Cunnington R, Windischberger C, Robinson S, Moser E (2006). The selection of intended actions and the observation of the others' actions: a time-resolved fMRI study. Neuroimage.

[B31] Molnar-Szakacs I, Kaplan J, Greefield P, Iacobini M (2006). Observing complex action sequences: the role of the fronto-parietal mirror neuron system. Neuroimage.

[B32] Taylor S, Stern E, Gehring W (2007). Neural systems for error monitoring: recent findings and theoretical perspectives. Neuroscientist.

[B33] Zaehle T, Jordan K, Wuestenberg T, Baudewig J, Dechent P, Mast FW (2007). The neural basis of the egocentric and allocentric spatial frame of reference. Brain Res.

[B34] Holroyd CB, Larsen JT, Cohen JD (2004). Context dependence of the event-related brain potential associated with reward and punishment. Psychophysiology.

[B35] Gehring WJ, Willoughby AR, Ullsperger M, Falkenstein M (2004). Are all medial frontal negativities created equal? toward a richer empirical basis for theories of action monitoring. Errors, Conflicts, and the Brain Current Opinions on Performance Monitoring.

[B36] McCarthy G, Wood CC (1985). Scalp distributions of event-related potentials: An ambiguity associated with analysis of variance models. Electroencephalogr Clin Neurophysiol.

[B37] Ruchkin DS, Johnson JR, Friedman D (1999). Scaling is necessary when making comparisons between shapes of event related potential topographies: a reply to Haig et al. Psychophysiology.

[B38] Menon V, Adleman NE, White CD, Glover GH, Reis AL (2001). Error-related brain activation during a Go/No Go response inhibition task. Hum Brain Mapp.

[B39] Krigolson OE, Holroyd CB (2007). Hierarchical error processing: different errors, different systems. Brain Res.

[B40] Kalaska JF, Crammond DJ (1995). Deciding not to go: neuronal correlates of response selection in a go/nogo task in primate premotor and parietal cortex. Cereb Cortex.

[B41] Nagahama Y, Okada T, Katsumi Y, Hayashi T, Yamauchi H, Sawamoto N, Toma K, Nakamura K, Hanakawa T, Konishi J, Fukuyama H, Shibasaki H (1999). Transient neural activity in the medial superior frontal gyrus and precuneus time locked with attention shift between object features. Neuroimage.

[B42] Freund HJ (2001). The parietal lobe as a sensorimotor interface: a perspective from clinical and neuroimaging data. Neuroimage.

[B43] Leiguarda RC (2003). Apraxias and the lateralization of motor functions in the human parietal lobe. Adv Neurol.

[B44] Decety J, Grezes J (1999). Neural mechanisms subserving the perception of human actions. Trends Cogn Sci.

[B45] Andersen RA, Buneo CA (2002). Intentional maps in posterior parietal cortex. Annu Rev Neurosci.

[B46] Shannon BJ, Buckner RL (2004). Functional-anatomic correlates of memory retrieval that suggest nontraditional processing roles for multiple distinct regions within posterior parietal cortex. J Neurosci.

[B47] Klein DN, Anderson RL, Miller GA (1995). The behavioral high-risk paradigm in the mood disorders. The behavioral high-risk paradigm in psychopathology.

